# The Influence of Floorball on Hematological Parameters: Consequences in Health Assessment and Antidoping Testing

**DOI:** 10.1155/2020/6109308

**Published:** 2020-07-27

**Authors:** Johan O. Wedin, Anders E. Henriksson

**Affiliations:** ^1^Department, Laboratory Medicine, Sundsvall County Hospital, Sundsvall, Sweden; ^2^Department of Natural Sciences, Mid Sweden University, Sundsvall, Sweden

## Abstract

Assessment of hematological parameters is common in sports medicine. Although physical exercise is an important preanalytical variable, data about acute hematological changes after high-intensity intermittent exercise are scarce. This study aimed to examine floorball as a potential preanalytical variable for hematological parameters used in health assessment and antidoping testing. Twenty-three professional male floorball players participated in a floorball game. Hematological parameters including hemoglobin, erythrocyte count and erythrocyte indices, reticulocytes, white blood cells (WBC), platelets, reticulocytes, and OFF-hr score were assessed at baseline, immediately postgame, and at 2 h postgame. Median hemoglobin concentration decreased significantly from 146 g/L pregame to 141 g/L immediately postgame (*p* < 0.001). WBC count increased from 7.2 × 10^9^/L pregame to 10.1 × 10^9^/L 2 h postgame (*p* < 0.001). The median OFF-hr score decreased from 99.5 to 94.2 immediately postgame and remained significantly lower than baseline at 2 h postgame (94.4, *p*=0.030). Looking at individual results, the highest OFF-hr score increased from 120 at baseline to 124 at 2 h postgame. Our findings suggest that participation in a floorball game affects several hematological parameters and consequently can affect health assessment and antidoping testing.

## 1. Introduction

Blood collection through venipuncture with subsequent laboratory analysis is a common medical procedure [1]. The complete blood count (CBC) with or without white blood cell (WBC) differential, a test that includes a battery of hematological parameters, is one of the most used blood tests in medical care [2].

The preanalytical phase is an essential component of laboratory quality, and several variables affect the result of a blood sample [3]. Physical exercise is a very important preanalytical variable as it causes acute alterations in a variety of laboratory parameters, including hematological parameters [4, 5]. These levels may fall outside conventional reference ranges used to diagnose pathological conditions [5, 6]. Consequently, this can affect health status interpretation of an athlete [7].

Hematological parameters also play another important role in sports medicine. CBC is frequently used in antidoping testing for the detection of blood doping [8]. The World Anti-Doping Agency (WADA) currently uses the Hematological Module of the Athlete Biological Passport (ABP) for indirect detection of blood doping, such as autologous blood transfusions or the use of erythropoiesis-stimulating agents [9]. Parts of the CBC, together with reticulocytes, are used to calculate the OFF-hr score, which is an index of hematopoietic stimulation. It can detect withdrawal as well as reinfusion of blood [10].

Floorball is a rapidly growing sport that contains episodes of high-intensity intermittent exercise of alternating aerobic and anaerobic character, and it can cause transient elevation of cardiac biomarkers [11, 12]. It was granted full International Olympic Committee (IOC) recognition at the 123^rd^ IOC meeting in Durban, South Africa, in 2011 and is a candidate for inclusion in the Olympic Games. Therefore, the International Floorball Federation has adopted the WADA antidoping code.

Few studies have examined the effect of high-intensity intermittent exercise on hematological and biochemical parameters [4, 5]. With an increasing number of floorball players worldwide, it is likely that players are subject to laboratory testing for both health assessment and antidoping testing. Floorball should, therefore, be assessed as a preanalytical variable. This study aimed to examine the effect of floorball on hematological and biochemical parameters. We hypothesized that several hematological and biochemical parameters would be influenced by a high-intensity sport.

## 2. Materials and Methods

### 2.1. Participants and Study Design

In this prospective, observational study, 23 male players from a professional floorball team participated in a floorball game, played in 3 × 20 minutes effective time (i.e., the time is stopped whenever there is a pause in the game), for evaluation of acute alterations in hematological parameters. The game was conducted in March 2014 in Sundsvall, Sweden, starting at 10 : 00AM. All players were competitive athletes from the same floorball team and had previously been screened for cardiovascular abnormalities. They were eligible for inclusion in the study if both ECG and echocardiogram were unremarkable. No player had taken any medications and no player had a history or illicit drug use. No player had donated blood or received blood transfusion, and there was no history of altitude training or simulation. All players were nonsmokers.

Oral and written information about the procedures was given before the study. Consent forms were obtained from all study participants and their parents/legal guardians if under 18 years. The study was approved by the regional ethics committee and all procedures complied with the ethical standards of the Declaration of Helsinki.

### 2.2. Blood Sampling

The participants refrained from exercise 24 h prior to the study to get adequate resting parameters. The study was conducted at sea level. Water intake was allowed *ad libitum* before, during, and after the game. Ingestion of food was not allowed from 2 h before the time of the first blood sampling until completion of the last blood sampling 2 h postgame. Blood was drawn at three time points: pregame (09 : 30AM), 0 h postgame (11 : 30AM), and 2 h postgame (01 : 30PM). Five players were also tested after 24 h to follow alterations for a longer time. Blood sampling was performed from an antecubital vein using a 21 G needle within 30 seconds of tourniquet application and under aseptic conditions. The players rested in a sitting position 10 minutes before and during each collection. Two experienced phlebotomists collected the samples.

Blood was collected into 3 mL vacutainer tubes containing K_2_-EDTA (BD Vacutainer®, Becton Dickinson, New Jersey, USA) to measure hematological parameters, and into 4 mL vacutainer tubes containing lithium heparin (BD Vacutainer®, Becton Dickinson, New Jersey, USA) to measure biochemical parameters. Subsequent to sampling, the tubes were mixed 10 times on a blood mixer, and all samples were immediately transported in an insulated cooler box to an accredited laboratory (SS-EN ISO/IEC 17025 and SS-EN ISO 15189) and analyzed within 30 minutes. No blood sample was discarded due to unsatisfactory venipuncture attempts.

### 2.3. Hematological Analyses

Laboratory testing was performed on calibrated instruments. Analytical quality was monitored by means and standard deviation of an internal quality control system and by participation in an external quality assurance program. Blood samples were inverted 20 times prior to analysis. Full blood count was determined using an automated hematology analyzer (Sysmex XE-5000, Kobe, Japan), according to the manufacturer's specifications. The CBC included hemoglobin (Hb), hematocrit (Hct), red blood cells (RBC), mean corpuscular volume (MCV), mean corpuscular hemoglobin (MCH), mean corpuscular hemoglobin concentration (MCHC), red cell distribution width (RDW-CV), platelet count (PLT), and leukocyte count including a quantitative differential count of lymphocytes, monocytes, neutrophils, eosinophils, and basophils. Reticulocyte count and percentage of reticulocytes were also obtained. No samples required manual review. The assays showed coefficients of variation (CV) < 5% for Hb, Hct, RBC, erythrocyte indices and reticulocytes, <6% for PLT, and <8% for leukocyte and differential count.

### 2.4. Biochemical Analyses

Blood samples were centrifuged for 7 minutes at 2000 ×*g* to separate plasma from blood cells. Plasma albumin, sodium, potassium, total calcium, creatinine, and lactate dehydrogenase (LD) were analyzed using assays from Roche Diagnostics on a fully automatic Cobas 6000 analyzer (Roche Diagnostics, Basel, Schweiz). Indices of hemolysis, icterus, and lipemia (HIL indices) were assessed for lithium heparin plasma to assure sample quality. The total CV was <5% for all assays.

### 2.5. Statistical Analyses

Data from all parameters were tested for normality using the Shapiro–Wilk test and verified by visual inspection of histograms. Normally distributed data are reported as mean and 95% confidence intervals (95% CI). Nonnormally distributed data are reported as median with interquartile range (IQR) values. The OFF-hr Score, which is an index of stimulation, was calculated as “Hb [g/L] – 60 ∗ √(Reticulocytes [%])”. At sea level, the normal range was 85–95, levels ≥104.6 require further investigation of an athlete, and levels ≥125.6 denote a start prohibition meaning that an athlete is not allowed to participate in any competitive events [13, 14]. The hematological and biochemical data are paired; i.e., they arise from the same individual at different points in time. ANOVA with repeated measurements was used to compare the findings between pregame and postgame values for normally distributed data. If the test was significant, contrasts were created for pairwise comparisons. In the case of Sphericity not assumed, it was corrected by Greenhouse–Geisser. The Friedman test was used to compare the findings between pregame and postgame values for nonnormally distributed data. If the test was significant, Dunn's pairwise post hoc test was used for further pairwise comparisons, where a Bonferroni correction was applied on the *p*-values. A *p-*value <0.05 was indicative of statistical significance. Statistical analyses were performed using IBM SPSS 26.0 (IBM, Armonk, NY, USA).

## 3. Results

Anthropometric measurements were conducted at enrollment, summarized in Table 1.

The body mass was measured in all players pregame and immediately postgame. The mean pregame body mass was 77.3 kg (72.8–81.8 kg). The mean body mass did not change significantly immediately postgame (77.1 kg, 95% CI 72.6–81.5 kg).

Levels of hematological parameters are presented in Table 2. The median Hb concentration decreased significantly from 146 g/L pregame to 141 g/L immediately postgame (*p* < 0.001) and returned to baseline (146 g/L) 2 h postgame. The median percentage difference pregame compared to 0 h postgame was 3.7% (IQR 1.5–4.7%). For the erythrocyte indices, only MVC were significantly altered immediately postgame and 2 h postgame compared to baseline. The mean WBC (leukocyte) concentration increased from 7.2 × 10^9^/L pregame to 8.6 × 10^9^/L (*p*=0.002) and 10.1 × 10^9^/L (*p* < 0.001) immediately postgame and 2 h postgame, respectively. Leukocyte differential count (Table 3) showed a significant increase in neutrophils, both 0 h postgame (*p* < 0.001) and 2 h postgame (*p* < 0.001). Also, monocytes increased significantly 0 h and 2 h postgame (*p*=0.029 and *p* < 0.001). There was a significant decrease in lymphocyte count 0 h postgame (*p*=0.010), and a decrease in eosinophils 0 h and 2 h postgame (*p* < 0.001 and *p*=0.003). No significant difference was shown after 24 h compared to baseline for Hb, Hct, RBC, leukocyte count, and PLT in five players tested.

No significant difference was shown immediately postgame and 2 h postgame compared to baseline for reticulocyte count. The percentage of reticulocytes displayed a nonsignificant increase from 0.70% to 0.75% immediately postgame, which returned to baseline 2 h postgame (0.70%).

Hb and percentage of reticulocytes are used for calculation of the OFF-hr score. The median baseline OFF-hr score was 100. The Median OFF-hr score decreased to 94 (*p* < 0.001) immediately postgame and remained significantly lower than baseline 2 h postgame (94, *p* < 0.030). Looking at individual results, the highest OFF-hr score was 120 at baseline, increasing to 124 at 2 h postgame. Three players with an OFF-hr score that was within the reference (85–95) pregame had levels below the normal range at 2 h postgame, and two players had levels >104.6 2 h postgame.

Biochemical parameters are presented in Table 4. Albumin was slightly decreased 2 h postgame (*p*=0.009) but not immediately postgame compared to baseline. There was no significant difference in levels of sodium and total calcium postgame compared to baseline. Potassium levels decreased from 3.9 mmol/L at baseline to 3.6 mmol/L at 0 h postgame (*p* < 0.001), normalizing to 3.7 mmol/L at 2 h were no statistical difference could be observed (*p*=0.137). Creatinine levels increased significantly from a median concentration of 84 µmol/L pregame to 94 *µ*mol/L immediately postgame (*p* < 0.001) and returned to 84 µmol/L 2 h postgame (*p*=0.279). Levels of LD were significantly lower at both 0 h (3.5 µcat/L, *p* = 0.010) and 2 h postgame (3.4 *µ*cat/L, *p*=0.015) compared to baseline (3.4 *µ*cat/L).

## 4. Discussion

In the present study, we assessed the impact on hematological parameters in 23 professional floorball players after a floorball game. Some variables included in the CBC were significantly altered after the game. These findings are, to our knowledge, the first observed after intermittent high-intensity exercise and have two important implications to be considered, primarily in sports medicine, hematology, and laboratory medicine.

First, with increasing participation in floorball, physicians and laboratory staff should acknowledge participation in a floorball game as a preanalyical variable altering several hematological parameters. CBC is assessed in clinical routine for screening and diagnosis of several disorders or to assess treatment progress and thus plays a significant role in clinical decision making [15]. Levels exceeding conventional reference ranges might, therefore, lead to disqualification without merit, unnecessary anxiety, extra medical costs, and even mistreatments. Thus, the preanalytical phase is fundamental for assuring correct interpretation of laboratory data. In the present study, the leukocyte count significantly increased postgame, probably due to endovascular demargination induced by glucocorticoids and augmented blood flow as suggested earlier [16]. As observed in previous studies, the leukocytosis was the result of increased numbers of neutrophils, while the other cell types remained stable throughout the observation period [17]. This type of leukocytosis should not be interpreted as infectious or inflammatory.

We found that Hb concentrations decreased significantly immediately postgame. It has been suggested to be the result of mechanically induced damage to the erythrocytes, known as mechanical hemolytic anemia [18]. However, this does not seem to be the explanation in this study as levels of lactate dehydrogenase decreased and reticulocyte count did not increase postexercise. Furthermore, plasma volume change contributes to the variability of many laboratory parameters [19], but generally, hemoconcentration is induced by intense exercise which would instead lead to increased Hb concentrations [20]. There are some investigations that found hypervolemia in the acute phase following intense exercise [21, 22]. One possible explanation for our finding is that hypovolemia rapidly induces a compensatory increase in blood volume by the exchange of fluid from the interstitium to the blood, which will result in hemodilution and thus decreased Hb concentration [23]. In support of this explanation, there was also a trend towards decreased levels of albumin and potassium in the present study. Since unlimited water intake was allowed, and no weight loss was observed, there was likely no net fluid loss. The hemodilution could instead be the result of an acute, relative hyperhydration. The Hb decrease was larger than the intraindividual biological variation of ±3% in 50% of the players (median decrease of 3.7%; IQR 1.5–4.7%) [23]. However, in most cases, this has no clinical relevance as only 2 of 23 players had levels at and just below the lower reference limit immediately postexercise.

The second important point is the impact on the hematological module of the Athlete Biological Passport (ABP) for antidoping testing, as limited data on how exercise affects ABP in the acute phase exist. There are mainly three types of blood doping, namely, blood transfusions, erythropoietin (EPO), and synthetic oxygen carriers. All methods enhance exercise performance in aerobic sports [24]. As there are several difficulties in direct testing of misuse, WADA introduced the ABP, where indirect markers have the potential to detect all types of blood doping on an individual level. The purpose of the Athlete Biological Passport is to detect abnormal variations by storing data on hemoglobin concentration and the percentage of reticulocytes over time so that an athlete is its own reference [25]. Several limitations with the ABP has been recognized; many of them are preanalytical factors including physical exercise [26].

Although the long-term effects of different types of exercise on hematological variables are well studied, data on the acute variation of Hb and reticulocytes induced by exercise are scarce [27]. In the present study, we found that percentage of reticulocytes was not significantly increased after the game. Thus, this would not alter the OFF-hr score. On the contrary, the Hb decrease postgame had an impact on the OFF-hr score calculation. As discussed above, the hemodilution could be the result of an acute, relative hyperhydration which was recently shown to reduce the OFF-hr score [27]. Although the median value of OFF-hr score decreased postgame, postgame levels increased in some players and exceeded an OFF-hr score of 104.6 after 2 h in two players. These players might be subject to a further investigation based on the variability of their results. The player with the highest postgame OFF-hr score of 124 was close to the start prohibition OFF-hr score at ≥125.6, and thus not far from being temporarily withdrawn from the competition without merit. It should be noted that each athlete is their own reference when it comes to antidoping testing, and the variations secondary to high-intensity exercise found in this study might not be completely negligible. Our findings are likely to not only be observed in floorball players but in other athletes participating in high-intensity sports as well. This needs to be further investigated.

Most players had false low OFF-hr score levels postgame concluding that intermittent high-intensity exercise acutely reduces the OFF-hr score significantly, thus reducing the sensitivity of the ABP hematological module. However, a fraction of players also had an increased OFF-hr score postexercise. There might be a risk of misinterpreting these physiological variations induced by exercise as false-positives, which would have drastic consequences for an individual athlete. According to the WADA rules, antidoping testing can be performed two hours after training or competition. Furthermore, it is stated that all testing is performed in the morning. Obviously, these rules are not always followed [3]. Thus, our study shows the importance of a precautionary marginal of more than 2 h for a more reliable antidoping evaluation. Future studies need to better define a safety marginal with more frequent sampling for a longer time-period postexercise.

Physicians in the field of laboratory medicine and hematology are likely to become involved in the evaluation of an athlete's hematological parameters and must be able to distinguish whether an abnormality is the result of an acute disorder, doping or a preanalytical error source. To assure analytical accuracy, floorball should be documented and referenced as a preanalytical variable when evaluating laboratory results in sports medicine.

Our study has some limitations to consider. The number of participants was small, and the general conclusions might not be applicable to a larger cohort. Another limitation is the timing of blood sampling. According to WADA guidelines, there should be no blood collection for 2 h following the competition. Although the 2 h postgame testing falls within these recommendations, we suggest that a future study of the same rationale should test different sampling times >2 h postexercise in order to test the validity of the ABP for a longer period postexercise. We did not analyze the blood samples twice as recommended, making our results somewhat uncertain.

## 5. Conclusion

In summary, we found that several hematological parameters are significantly altered after a floorball game in professional players. This can have serious consequences for health assessment and antidoping testing. It is important that an athlete serves as its own reference, as conventional reference intervals and cutoffs might not be appropriate.

## Figures and Tables

**Table 1:** Baseline characteristics of the 23 floorball players included in the study.

Age, years	20 (18–23)
Height, cm	183 (181–186)
Body mass, kg	77.3 (72.8–81.8)
BMI, kg/m^2^	22.9 (21.9–23.8)

Numerical data are presented as mean (95% CI).

**Table 2:** Changes in hematological parameters in 23 floorball players after the floorball game.

Assay, reference limit	Baseline	0 h	2 h
*Normal distribution*			
RBC, 4.25–5.71 × 10^12^/L	4.91 (4.81–5.01)	4.77 (4.67–4.88)^*∗∗∗*^	4.81 (4.71–4.92)^*∗∗∗*^
MCH, 27.1–33.3 pg	30.1 (29.6–30.6)	30.0 (29.5–30.5)^NS^	30.1 (29.5–30.6)^NS^
MCHC, 317–357 g/L	341 (338–345)	342 (339–345)^NS^	343 (339–347)^NS^
MCV, 82–98 fL	88.2 (87.0–89.5)	87.6 (86.4–88.9)^*∗∗∗*^	87.7 (86.5–89.0)^*∗∗*^
RDW-CV, 12.3–14.3%	12.8 (12.7–13.0)	12.8 (12.6–13.0)^NS^	12.8 (12.6–13.0)^NS^
PLT, 145–348 × 10^9^/L	245 (227–262)	246 (224–267)^NS^	249 (230–267)^NS^
WBC, 4–11 × 10^9^/L	7.2 (6.5–8.0)	8.6 (7.7–9.4)^*∗∗*^	10.1 (9.3–11.0)^*∗∗∗*^

*Nonnormal distribution*			
Hb, 134–170 g/L	146 (144–151)	141 (138–146)^*∗∗∗*^	146 (139–149)^*∗∗∗*^
HCT, 40–50%	43 (42–44)	41 (41–42)^*∗∗∗*^	42 (41–43)^*∗∗∗*^
RET, 29–89 × 10^9^/L	39.4 (29.2–43.6)	38.6 (27.8–41.1)^NS^	36.7 (29.1–41.7)^NS^
RET%, 0.5–1.5%	0.70 (0.59–0.87)	0.75 (0.60–0.90)^NS^	0.70 (0.61–0.87)^NS^
OFF-hr score, 85–95	99.5 (88.0–102.6)	94.2 (84.4–97.4)^*∗∗∗*^	94.4 (82.9–100.5)^*∗*^

Normally distributed values are expressed as mean (95% CI) and nonnormally distributed values are expressed as median (IQR). RBC: red blood cells; MCH: mean cell hemoglobin; MCHC: mean corpuscular hemoglobin concentration; MCV: mean corpuscular volume; RDW-CV: red cell distribution width; PLT: platelets; WBC: white blood cell (leukocyte) count; Hb: hemoglobin; HCT: hematocrit; RET: reticulocyte count; RET%: percentage of reticulocytes. ^NS^nonsignificant, ^*∗*^*p* < 0.05, ^*∗∗*^*p* < 0.01, and ^*∗∗∗*^*p* < 0.001compared to baseline.

**Table 3:** Leukocyte differential count in 23 floorball players before and after the floorball game.

Assay, reference limit	Baseline	0 h	2 h
Lymphocytes, 0.9–3.0 × 10^9^/L	2.45 (2.18–3.02)	2.13 (1.73–2.57)^*∗*^	2.53 (2.22–3.20)^NS^
Neutrophils, 1.8–7.4 × 10^9^/L	3.38 (2.76–4.19)	5.08 (3.97–6.62)^*∗∗∗*^	5.91 (5.08–7.29)^*∗∗∗*^
Eosinophils, 0.0–0.4 × 10^9^/L	0.15 (0.09–0.23)	0.11 (0.06–0.16)^*∗∗∗*^	0.09 (0.05–0.16)^*∗∗*^
Basophils, 0.0–0.1 × 10^9^/L	0.02 (0.02–0.03)	0.02 (0.02–0.04)^NS^	0.02 (0.02–0.05)^NS^
Monocytes, 0.2–0.8 × 10^9^/L	0.61 (0.51–0.68)	0.67 (0.54–0.85)^*∗*^	0.83 (0.64–1.03)^*∗∗∗*^

Data are expressed as median (IQR). ^NS^nonsignificant, ^*∗*^*p* < 0.05, ^*∗∗*^*p* < 0.01, and ^*∗∗∗*^*p* < 0.001 compared to baseline.

**Table 4:** Changes of biochemical parameters in 23 floorball players before and after a floorball game.

Assay, reference limit	Baseline	0 h	2 h
Albumin, 36–48 g/L	49 (48–50)	49 (48–50)^NS^	48 (47–49)^*∗∗*^
Sodium, 137–145 mmol/L	142 (142–143)	142 (142–143)^NS^	142 (141–142)^NS^
Potassium, 3.5–4.4 mmol/L	3.9 (3.8–4.0)	3.6 (3.4–3.7)^*∗∗∗*^	3.8 (3.6–3.9)^NS^
Total calcium, 2.15–2.50 mmol/L	2.26 (2.23–2.30)	2.28 (2.25–2.31)^NS^	2.25 (2.23–2.28)^NS^
Creatinine, 60–105 *µ*mol/L	86 (81–91)	94 (89–99)^*∗∗∗*^	84 (81–88)^NS^

Values are expressed as mean (95% CI). ^NS^nonsignificant, ^*∗∗*^*p* < 0.01, and ^*∗∗∗*^*p* < 0.001 compared to baseline.

## Data Availability

The data used to support the findings of this study are available from the corresponding author upon request.
